# Resonant Akhmediev breathers

**DOI:** 10.1038/s41598-024-61533-1

**Published:** 2024-05-09

**Authors:** Amdad Chowdury, Dawn T. H. Tan

**Affiliations:** 1 Photonics Devices and Systems Group, Singapore University of Technology and Design, 8 Somapah Rd., Singapore, 487372 Singapore; 2 Institute of Microelectronics (IME), Agency for Science, Technology and Research (A*STAR), 2 Fusionopolis Way, Innovis #08-02, Singapore, 138634 Singapore

**Keywords:** Applied mathematics, Statistical physics, thermodynamics and nonlinear dynamics, Nonlinear optics, Solitons

## Abstract

Modulation instability is a phenomenon in which a minor disturbance within a carrier wave gradually amplifies over time, leading to the formation of a series of compressed waves with higher amplitudes. In terms of frequency analysis, this process results in the generation of new frequencies on both sides of the original carrier wave frequency. We study the impact of fourth-order dispersion on this modulation instability in the context of nonlinear optics that lead to the formation of a series of pulses in the form of Akhmediev breather. The Akhmediev breather, a solution to the nonlinear Schrödinger equation, precisely elucidates how modulation instability produces a sequence of periodic pulses. We observe that when weak fourth-order dispersion is present, significant resonant radiation occurs, characterized by two modulation frequencies originating from different spectral bands. As an Akhmediev breather evolves, these modulation frequencies interact, resulting in a resonant amplification of spectral sidebands on either side of the breather. When fourth-order dispersion is of intermediate strength, the spectral bandwidth of the Akhmediev breather diminishes due to less pronounced resonant interactions, while stronger dispersion causes the merging of the two modulation frequency bands into a single band. Throughout these interactions, we witness a complex energy exchange process among the phase-matched frequency components. Moreover, we provide a precise explanation for the disappearance of the Akhmediev breather under weak fourth-order dispersion and its resurgence with stronger values. Our study demonstrates that Akhmediev breathers, under the influence of fourth-order dispersion, possess the capability to generate infinitely many intricate yet coherent patterns in the temporal domain.

## Introduction

The most evolutionary physical system that has many internal interacting components or agents deviates from their initial equilibrium state over time and can develop instability in the system. A particular type of instability, namely, modulation instability (MI), arises in many areas of physics including but not limited to hydrodynamics^1–3^, nonlinear optics^4^, plasma physics^5^, biophysics^6^, nonlinear self-organization and pattern formations^7^.

In nonlinear optics, MI remains at the heart of many nonlinear optical phenomena that arise when light propagates through a nonlinear optical medium such as crystal, optical fiber, or waveguides. Noise, which is naturally present in the applied optical field seeds the instability which upon further propagation amplifies exponentially due to its interaction with the dispersion and nonlinear properties of the medium. In the frequency domain this is equivalent to generating cascades of spectral sidebands^8,9^. In the more developed stage of MI inside the medium, the dynamics are highly complex and involve several stages of energy exchange among the spectral modes. This process is intimately connected with a novel nonlinear phenomenon called the Fermi-Pasta-Ulam (FPU) recurrence^10,11^.

In an optical fiber, the FPU recurrence is: when the modulated input pump starts to propagate in the fiber, the pump generates new sidebands by giving up its own energy to these sidebands. When all the energy from the pump is transferred to many of the generated sidebands, we see the AB just reached its highest amplitude. However, the process starts to reverse at this point when the AB starts to come down from the highest amplitude point. The pump starts to take back its energy from the sidebands and eventually returns to its initial state where it started in the first place - which is the recurrence. This completes a growth-return cycle of an AB and is called the FPU recurrence.

Applying the nonlinear Schrödinger equation (NLSE), early research on MI and FPU recurrence was done mainly using numerical studies. In 1984, Hasegawa first showed that one can generate a series of short optical pulses with a desired repetition rate with a limited number of initial conditions^12^. The following year, Akhmediev et al. developed a generalized theory and gave a solid mathematical foundation to the description of MI by deriving the exact analytical solution namely, the Akhmediev breather (AB) presented in^13^.

The defining physics underpinning the development of an AB through MI is to excite a pair of sidebands on both sides of the input optical pulse. The subsequent dynamics follow a four-wave mixing process (FWM)^14^ and generate cascades of new modulation frequencies in a triangular fashion which are harmonics of the initial pair of MI frequencies^15^. The end product is a full-grown AB forming a series of pulses in the time domain. Considerable attention has been paid to studying ABs not only because it can precisely describe MI, but later research revealed that the solution is also connected to a much-debated topic of ‘rogue waves’ - an unusually high amplitude wave that appears in many physical systems such as nonlinear optics and hydrodynamics^16,17^. While AB plays a significant role in demystifying the appearance of oceanic rogue waves^18^, the first optical rogue wave experimentally realized in fiber supercontinuum generation was reported in 2007^19^.

Apart from MI, the dispersive properties of the media are central in the field of nonlinear optics. They have been extensively studied in connection with several key novel nonlinear phenomena such as dispersive wave generation, soliton fission, and supercontinuum generation^14^. Manipulation of higher-order dispersion in optical fibers and waveguides is now a vital tool to generate new frequencies by transferring energy into inaccessible wavelength regimes^20,21^. Among the higher-orders of dispersion, third-order dispersion (TOD) has been found to play a dominant role in connection with MI, Cherenkov radiation, and FPU recurrence^22–24^.

However, the rapid progress in fiber drawing and manufacturing together with achieving greater freedom in controlling the dispersion curve both in normal and anomalous dispersion regimes strongly demands a careful look at higher-order dispersion $> TOD$ and beyond^20^. It is also important to note that, in the last several decades while extraordinary progress has been made in the area of optical fiber communication systems, with the advent of new material designs and dispersion engineering, state-of-the-art fibers and waveguides are emerging. Particularly, in the area of integrated photonics, complementary- metal-oxide-semiconductor (CMOS) based waveguides are becoming an attractive platform to demonstrate novel nonlinear phenomena^25–28^. These waveguides harness a large nonlinearly and dispersion on a very short length scale of a few mm where higher-order dispersion plays a dominant role. Hence, to reveal their dispersion landscape and light-wave behavior, studying the impact of even higher-order dispersion is crucial to utilize their potential for application.

The next higher-order dispersion, which is fourth-order dispersion (FOD) is studied in various contexts both theoretically and experimentally in nonlinear optics^29–35^. Many of these studies only partially address its impact on phase matching and MI dynamics. Abdullaev et al. first noted that the negative second-order dispersion (in anomalous dispersion regime) together with positive FOD (in normal dispersion regime) introduces a new area of MI and observed FPU recurrence^36^. However, there is little to no study in connection with AB. A clear and comprehensive study of FOD describing MI and its precise connection with AB, resonant radiation, or Cherenkov radiation both in the temporal and spectral domains is yet to be done.

This study offers a thorough explanation of the underlying physical principles behind how FOD within the normal dispersion regime triggers resonant radiation via MI. We demonstrate that FOD significantly impacts the evolution of an AB, primarily through resonant radiation-driven MI. Furthermore, we provide an in-depth analysis of the intricate energy exchange processes involved in these interactions. By employing precise mathematical formulations, we elucidate the phenomenon of AB disappearance with moderate positive FOD values, followed by its reemergence with stronger values.

In “Region of modulation instability” , we find the region defined by frequency and FOD parameter where MI exists. Only within this region, MI will induce the development of an AB. Each point within this region corresponds to one MI frequency and that will excite an AB for one FOD value. “Results”  describes the MI region where strong resonant interactions take place whereas “Energy exchange among the harmonics in the resonant MI interaction regime”  explains the energy exchange processes in these interactions. Finally, in “[Sec Sec6]”  we show the properties of the extended evolution of ABs while they undergo resonant MI development. In this section, we track the trajectory of the maximas of the AB along the evolution direction and illustrate how resonant interactions disrupt the AB development and again allow the reappearance of an AB.

## Region of modulation instability

The NLSE is the widely used equation that can capture the properties of long optical pulse propagation inside a fiber. However, with increasing pulse power, the fiber triggers varieties of higher-order linear and nonlinear optical effects such as higher-order dispersion, optical shock, and Raman effects^14^. With these effects, the fundamental NLSE is unable to model the propagation dynamics and needs modification. Incorporating these effects in NLSE the generalized nonlinear Schrödinger equation (GNLSE) is formed. Using the GNLSE, the first step towards studying the MI in a fiber system is to conduct a stability analysis using a propagating continuous wave to find the nature of MI in the system (see Methods section). We can represent the boundary of the region denoting the presence of MI for $\beta _4>0$ with the following pair of curves:

1 $$\begin{aligned}{} & {} \beta _4{(1)} = \frac{12}{\omega ^2}\,\,\, \text {and}\,\,\,\beta _4{(2)}=\frac{12 \left( \omega ^2-4\right) }{\omega ^4} \end{aligned}$$

**Figure 1:**
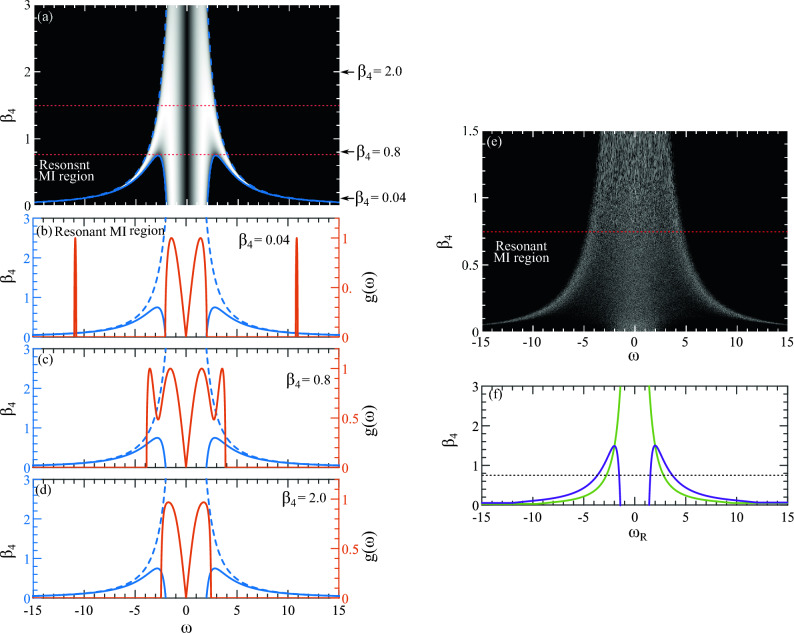
The relationship between the $\beta _4$ coefficient and the MI frequency $\omega$ is examined under different conditions. In (**a**), the MI frequency range is depicted for all $\beta _4 > 0$, with $\beta _2 = -1$. The bright region indicates the presence of MI, while the dark region represents its absence. Three distinct MI regions are identified and separated by red-dotted lines. (**b**) Illustrates the MI band within the resonance MI region with a solid brick-red curve for $\beta _4 = 0.04$, denoted by a black arrow in (**a**). Similarly, (**c**) shows the region of MI where two MI frequencies are within the same MI band, and (**d**) shows the MI band with one MI frequency only, with the corresponding positions highlighted in (**a**) using black arrows. The right Y-axis represents the growth rate $g(\omega )$, and the blue curves (Eq. 1) represent the upper (dashed) and lower (solid) boundaries on the $(\beta _4, \omega )$ plane where MI occurs. The left Y-axis displays $\beta _4$. (**e**) Similar to (**a**) but numerically generated using a white noisy initial condition (Eq. 2). (**f**) Presents only those modulation frequencies with the highest gain, based on Eqs. (3) and (4), depicted by purple and green curves. Below the dotted black line marks the resonant MI regimes (please see the Supplementary Material to see the impact where a movie is also provided with varying $\beta _4$ vs $\omega$).

In Fig. 1a, the region indicating the presence of MI is highlighted in the bright area for all $\beta _4 > 0$, with the upper limit plotted up to $\beta _4 = 3$. In Fig. 1b, the upper dashed blue curve represents $\beta _4(1)$, while the lower solid curve represents $\beta _4(2)$, enclosing the region where MI frequencies exist. The *resonant MI region* is defined within $0 < \beta _4 \le 0.75$. The corresponding MI band is presented in Fig. 1b with brick red lines. The primary MI band is situated in the central region between $\omega = -2$ and $2$ in Fig. 1a, where a narrow bandwidth MI curve emerges at the wings for small values of $\beta _4$. The position of the MI band is marked by the black arrow at the bottom of Fig. 1a. As $\beta _4$ increases, this narrow band gain curve converges and eventually merges with the central region.

If an AB development is initiated by MI frequency from the resonant MI region, the MI dynamics here are predominantly influenced by phase-matching interactions. This behavior mirrors the well-explored phenomenon of soliton dispersive wave generation, where the soliton’s wavenumber aligns with that of a linear wave, satisfying a phase-matching condition, resulting in energy leakage from the soliton in the form of dispersive waves^24^.

Note that the phenomena of MI and phase-matching have an intimate relationship where the former is a process, and the latter is a condition required for optimal MI. In this work, the resonant MI dynamics refers to the interaction between ABs that arise from two separate MI bands where both MI and phase-matching mechanisms play a key role in the dynamic processes. In resonant MI dynamics, phase-matching takes place in two stages. In the first stage, during the development of the ABs, the phase-matching condition must be satisfied to spontaneously generate the discrete spectral sidebands from the noise^14^ (see Sect. 10.2) that can achieve gain. It does not have any counterparts in general soliton dynamics. The second stage of phase matching takes place at the maximum compression point of the ABs when their spectrum overlaps with dispersive linear waves which have a direct counterpart in soliton phase matching phenomena arising from higher-order dispersion^24^.

In the succeeding MI region within the range $0.75 < \beta _4 \le 1.5$, the narrow-band MI curve at the wings converges and becomes part of the central wide-band MI curve. One example of the corresponding MI curve in this region is presented in Fig. 1c with $\beta _4=0.8$ . Unlike the preceding case, in this region, MI is characterized by a double-peak gain band. The perturbation frequency with the highest gain corresponds to the first peak and the second peak represents another harmonics of the first peak with the highest gain. Interaction between perturbation frequency and its harmonic within one gain band is defined as higher-order MI^37^. Finally, in the uppermost region ($\beta _4 > 1.5$) only one MI band is situated in the central area. An example of a corresponding MI band with $\beta _4 = 2.0$ is presented in Fig. 1d. Here, the AB develops from a perturbation frequency from the unstable gain band predicted by the linear stability analysis. Further elucidation of the impact of each scenario on an AB will be provided in subsequent sections.

To investigate if the similar MI region as shown in Fig. 1a can naturally arise in a waveguide, we employ a white-noise initial condition:

2 $$\begin{aligned} \psi (z=0,t) = 1+a(t) +i\,b(t) \end{aligned}$$

in Eq. (7), where $a(t)$ and $b(t)$ are two independent real random functions with values uniformly distributed around $0$. Numerical results indeed demonstrate that the MI frequencies observed in Fig. 1a are excited in Fig. 1e. As the initial white noise propagates inside the waveguide, the frequency component corresponding to the highest gain in the noisy initial condition undergoes exponential amplification. We simulate the evolution over a distance of $z=20$, which proves sufficient to excite enough frequencies with the highest gain.

For each value of $\beta _4$ in Fig. 1e, the excited frequency curve appears with roughness on its profile. When MI is excited with noise initially, the growth of the associated ABs becomes chaotic. This results in the development of a continuous spectrum around the MI frequencies with the highest gain, accompanied by noisy spectral content, as illustrated in Fig. 1e. This scenario mirrors realistic conditions encountered in waveguides or fibers.

Conversely, when an MI is excited with an exact AB solution as the initial condition, the resulting spectra are discrete due to the exact periodicity of the ABs and are devoid of noise. In such cases, only the frequencies with the highest gain are stimulated. While this scenario is idealized, it does not always reflect practical situations in waveguides and fibers.

It is noteworthy that Fig. 1a, derived from an exact stability analysis expression, demonstrates the absence of MI at approximately $\omega =0$ for all $\beta _4$. However, when stimulated with noisy initial conditions as depicted in Fig. 1e, this region becomes filled with spurious excitation of frequency components. Nevertheless, utilizing the exact AB solution remains a crucial tool for systematically investigating AB behavior.

To obtain a smoother profile, we simulate $1500$ values of $\beta _4$ ranging from $0$ to $1.5$. Remarkably, when put together a large number of $\beta _4$ vs $\omega$ profiles, the generated MI region closely aligns with the corresponding analytical case depicted in Fig. 1a. The frequencies $\omega$ that achieve the maximum growth rate for each $\beta _4$ are given by:

3 $$\begin{aligned} \omega _{R1} = \pm \sqrt{q_1/\beta _4}, \,\,\,\,\,\,\,\,\, \omega _{R2} = \pm \sqrt{q_2/\beta _4} \end{aligned}$$

where $q_1 = 6 - 2\sqrt{9-6\beta _4}$ and $q_2 = 6 + 2\sqrt{9-6\beta _4}$. $\omega _{R1}$ and $\omega _{R2}$ are the MI frequencies from the two sub-bands that have the maximum growth rate. This relationship is depicted by the purple curve in Fig. 1d. Equation (3) indicates that, for every $\beta _4$ value, the gain curve (purple) exhibits four symmetrical maxima on both sides of $\omega = 0$ until $\beta _4 < 1.5$. These maximas are divided into two groups, such as frequencies within the range $0 < \beta _4 \le 0.75$ participate in *resonant MI* dynamics, while those within $0.75 < \beta _4 \le 1.5$ initiate higher-order MI where MI frequencies are within the same MI band. Beyond $\beta _4 > 1.5$, the highest gain MI frequencies indicate MI with a single MI frequency. Notably, at $\beta _4 = 3$, the growth rate $g(\omega ) = \pm \sqrt{2}$ deduced from Eq. (12) aligns with the standard MI frequency derived from the conventional AB solution^13^. Also, for each $\beta _4$ there exists at least one frequency on the growth curve $g(\omega )$ where MI is zero and these points are given by:

4 $$\begin{aligned} \beta _4^{R}{(2)} =\frac{6}{\omega ^2} \end{aligned}$$

which is presented by the green curve in Fig. 1d. The width of both MI bands can be expressed by simple formulas. The two endpoints of the narrow gain band occurring at the wings are given by:

5 $$\begin{aligned} \omega _{b_1}= 2[3+\sqrt{(9-12\,\beta _4)}] \,\,\,\text {and} \,\,\,\omega _{b_2}=2\sqrt{\frac{3}{\beta _4}} \end{aligned}$$

The bandwidth of this gain band, denoted by $\Delta \omega _b=\omega _{b_2}-\omega _{b_1}$, represents the range of frequencies within it. On the other hand, the bandwidth of the central gain band always spans from $0$ to $\pm 2[3-\sqrt{(9-12\,\beta _4)}]$. By varying the value of $\beta _4$, the bandwidth can be determined for the MI frequencies within the three specific regions defined in Fig. 1a.

Until now, our discussion has centered on understanding the nature of MI in the presence of FOD. However, to comprehend how different types of MI regions affect an AB solution, it is essential to numerically generate an AB. While white noise initial conditions can yield AB-like structures during evolution, they tend to be highly chaotic, making it challenging to provide a clear explanation of MI in the presence of $\beta _4$. The standard analytic AB solutions can precisely explain MI only when $\beta _2 = -1$, a scenario that excludes higher-order dispersion terms. In this study, our objective is to investigate the impact of arbitrary values of $\beta _4$ on AB. To achieve this, we numerically solve Eq. (7) with a more accurate initial condition:

6 $$\begin{aligned} \psi (z=0,t) = 1+\alpha _\text {mod}\, \text {cos}(\omega \,t) \end{aligned}$$

Here, $\alpha _\text {mod}$ is a small real number representing the magnitude of modulation, and $\omega = \omega _{R1}$ is the modulation frequency from the central MI band with the maximum gain which facilitates the formation of an AB. As the initial wave propagates along the $z$-direction, multiple instances of AB emerge and recur due to the Fermi-Pasta-Ulam (FPU) recurrence mechanism, as detailed in^23^. The AB solution effectively characterizes both the MI and FPU processes. Given its comprehensive coverage in prior works^4,15,37–40^, we refrain from reiterating it here. In the subsequent sections, we explore how an AB is influenced by the various MI regions outlined in Fig. 1a.

## Results

## Impacts of $\beta _4$ on an Akhmediev Breather in the resonant MI region

**Figure 2:**
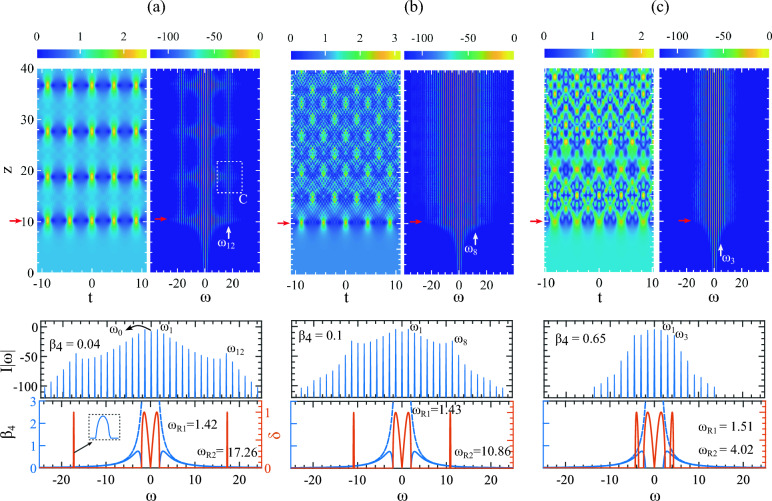
Impacts of $\beta _4$ on an AB in the resonant MI region. In each case (**a–c**), the top-panel shows the temporal and spectral evolution while the mid-panel shows the resonant amplification of the harmonics of the AB’s discrete spectrum. The bottom-panel shows the movements of the MI band compared to its region boundary. The discrete spectrums are taken at AB’s first compression points indicated by the red arrows with the values of (**a**) $\beta _4 = 0.04$, (**b**) $\beta _4 = 0.1$, and (**c**) $\beta _4 = 0.65$. In each case, the MI frequency $\omega _{R1}$ excites the first harmonic $\omega _1$ and $\omega _{R2}$ excites the phase-matched harmonics in (**a**) $\omega _{12}$, (**b**) $\omega _8$ and (**c**) $\omega _3$.

In the resonant MI region, we start perturbing the standard AB with $\beta _4 = 0.04$. In the evolution field, the initial condition Eq. (6), develops an AB that closely matches with the exact breather solution, at least the first appearance in Fig. 2a^38^. The development of the AB is initiated by the excitement of MI frequency $\omega _{R1} = 1.42$ from the central MI gain region as shown in the bottom panel at Fig. 2a. The boundary of the MI region is shown with blue and dashed solid lines.

After the AB is fully developed with perturbation frequency $\omega _{R1} = 1.42$, at the maximum compression point, it resonantly and symmetrically excites the second MI frequency $\omega _{R2} = 17.26$ which falls under the outside MI bands at the wings. Following Eq. (3), this arises as the $12^{th}$ harmonics of $\omega _{R1}$ as $\omega _{12}$ shown in the mid-panel where the pump $\omega _0$ is shown with the black arrow and the first sideband $\omega _1=\omega _{R1}$. Note that $\omega _{12}$ is phase-matched with the dispersive wave generated at this frequency and always arises only when the AB is excited by $\omega _{R1}$ defined by Eq. (3). We shall see later that the strength of the phase-matched energy exchange among the spectral components significantly influences the ABs’ extended temporal evolution.

After a phase-matched excitation of $\omega _{12}$, it starts a highly complex energy exchange process among the neighboring discrete frequency components along *z*. Note that $\omega _{12}$ gets excited at the first compression point of the breather at $z\approx 10$ indicated by the red arrow. It (white vertical arrow in the frequency domain), acts like a second pump besides $\omega _0$ and can generate sidebands similar to the pump. One of the side recurrences that arise from $\omega _{12}$ is indicated by the white box on the frequency evolution on the top panel at $z \approx 19$. It is produced by the main AB formed at $z \approx 10$ and arises due to spectral overlapping of the broadened main AB spectrum. We stress here that the first perturbation frequency is defined by Eq. (3) which is $\omega _{1} = \omega _{R1} = 1.43$ and the phase-matched $12^{th}$ harmonic arises at $\omega _{12} = 17.26$. A small offset of excitation frequency at $\omega _{12}$ arises due to the fact that the solution is derived by numerical simulation which can deviate slightly from the exact value. After excitation, the small side recurrences interfere with the main AB, creating dispersive linear waves in the background in both directions.

With $\beta _4=0.04$, the strength of the interference among the generated dispersive waves is low enough that the main AB and its recurrences are still sustained. For all the values with $\beta _4<< 0.75$, we observe these spectral and temporal dynamics where we show only one example in Fig. 2a. We will devote a separate section to discuss a more detailed picture of this scenario.

With an increase of $\beta _4$ the external MI band is coming closer to the pump with widening bandwidth as shown in Fig. 2b with $\beta _4 = 0.1$. The primary sideband next to pump $\omega _0$ is excited at $\omega _{R1}= 1.43$ as the first harmonic $\omega _1$ shown in the bottom panel. The notable feature in the top panel is the formation of a highly complex temporal pattern with more amplified wide-bandwidth frequency components. Also, another notable feature is after its first appearance at $z\approx 10$, the AB lost its recurrence property entirely. The Presence of highly amplified dispersive waves generated by the phase-matched $\omega _{R2} = 10.86$ at the AB’s $\omega _8^{th}$ harmonic dominates the background hindering the resurgence of the AB in the extended evolution. A detailed characterization of this behavior is provided in “Energy exchange among the harmonics in the resonant MI interaction regime”  in Fig. 5d–f in the spectral domain and “[Sec Sec6]”  where we track the evolution trajectory of the AB (see Fig. 7b).

Comparing the spectral intensity of $I|\omega |$ for $\beta _4 = 0.04$ with $\beta _4 = 0.1$ clearly shows that the spectrums are amplified more in the latter case. The linear dispersive waves that arise symmetrically at the first compression point of the AB are strong enough to disrupt or destroy altogether the possibility of the next appearance of the AB. This is shown in the temporal domain where the interference among the linear waves creates a coherent pattern along *z*.

**Figure 3:**
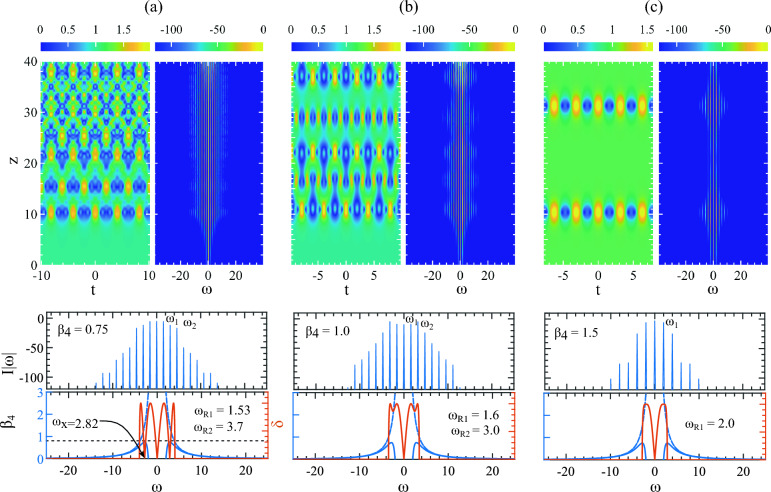
Impact of strong FOD on a numerically excited AB in the MI region $0.75 < \beta _4 \le 1.5$ where the MI frequencies $\omega _{R1}$ and $\omega _{R2}$ remain close to each-other. Three instances of evolution with (**a**) $\beta _4 = 0.75$, (**b**) $\beta _4 = 1.0$ and (**c**) $\beta _4 = 1.5$ are shown. With $\beta _4=0.75$, both MI-bands come in contact at $\omega _x=2.82$ where there is no MI. With an increasing value of $\beta _4$, the recurrence dynamics are returning to their regular behavior. In (**c**) the MI gain band resolved into perfect AB recurrence dynamics where only one MI frequency develops one standard AB.

With a further increase of $\beta _4$ to 0.65, the outside MI band is much closer to the central MI band with a slightly wider bandwidth shown in the bottom panel of Fig. 2c. The first sideband $\omega _1$ excited at $\omega _{R1}= 1.51$ and the second phase-matched harmonic $\omega _3$ excited at $\omega _{R2}= 4.02$. The generated AB’s bandwidth is narrower than before. It is worthwhile to mention that the spectral width depends on the position of the narrow MI band. If this band is far away from the pump, the AB excites all the harmonics until it excites the harmonics which is within the narrow MI band. With stronger $\beta _4$, this band appears close to the pump, hence, the AB has to excite fewer harmonics before it can be phase-matched with the harmonic within the narrow MI band.

However, the intensity of the spectrums near the pump is even higher as shown in the middle panel. With this, the interference among generated linear waves is even stronger albeit with fewer amplified frequencies resulting in the complex temporal evolution creating a coherent pattern shown in the top panel. Notably, the first appearance of AB at $z\approx 10$ is highly modulated due to the strong interference that arises from the dispersive wave generated by the phase-matched harmonic frequency $\omega _3$ with $\omega _1$ and $\omega _2$. Also, the patterns on the background are qualitatively different from those formed in Fig. 2b. A strong resonant interaction among the ABs seeded by the phase-matched harmonics takes place only when $0<\beta _4 \le 0.75$. It appears that there exists a correlation between the number of excited harmonics and their strength with the formed structures, and their complexity. A detailed explanation in this direction is beyond the scope of this current manuscript but can be the subject of future study and analysis.

We find that when $\beta _4=0.75$ the MI band (brick-red curve) in Fig. 3a develops a cusp at $\omega _x = 2 \sqrt{2} = 2.82$ and excits $\omega _{R1} = 1.53$ and $\omega _{R2} = 3.7$ at their maximum gain. The MI cannot develop at cusp point $\omega _x$. Note that it also marks the last point where the external and the central MI bands are separate from each other. After this point with $\beta _4>0.75$, the external MI band starts to merge with the central MI band. Now we have one perturbation frequency and a phase-matched harmonic defined by $\omega _{R1}$ and $\omega _{R2}$ respectively. A double peak MI band characterizes them and they excite two mutually interacting AB.

In this regime of MI, the development of an AB is dominated by higher-order MI dynamics where the resonant interaction is reduced significantly. This begins with the appearance of the lost AB again which is obscured previously by strong resonant MI interactions. In Fig. 3a, in the temporal domain, we can see hints of the return of the recurrence cycles. Two complete growth-return cycles take place at the early stage of the evolution.

When MI bands are even closer to each other with increasing $\beta _4$, the regular behavior of the recurrence cycle of an AB also starts to restored to its full shape. Figures 3b, c show the ABs evolution for $\beta _4 = 1.0$ and 1.5. In the top panel, on the frequency evolution, the repeated cycle of compression and decompression stages appeared clearly. With $\beta _4 = 1.0$ two MI frequencies within the same band are $\omega _{R1} = 1.6$ and $\omega _{R2} = 3.0$. In these cases, the dynamics of higher-order MI dynamics are at play resembling the MI dynamics presented in^37^, (see Figs. 1 and 2).

However, only at $\beta _4 = 1.5$, the external MI band completely merges with the central MI band marking the end of the resonant MI dynamics regime. With $\beta _4\ge 1.5$, the MI band possesses only one gain band with one maxima restoring the regular growth return cycle of a standard AB which is highlighted in Fig. 3c (please see the Supplementary Material to see these dynamics in a movie).

## Energy exchange among the harmonics in the resonant MI interaction regime

**Figure 4:**
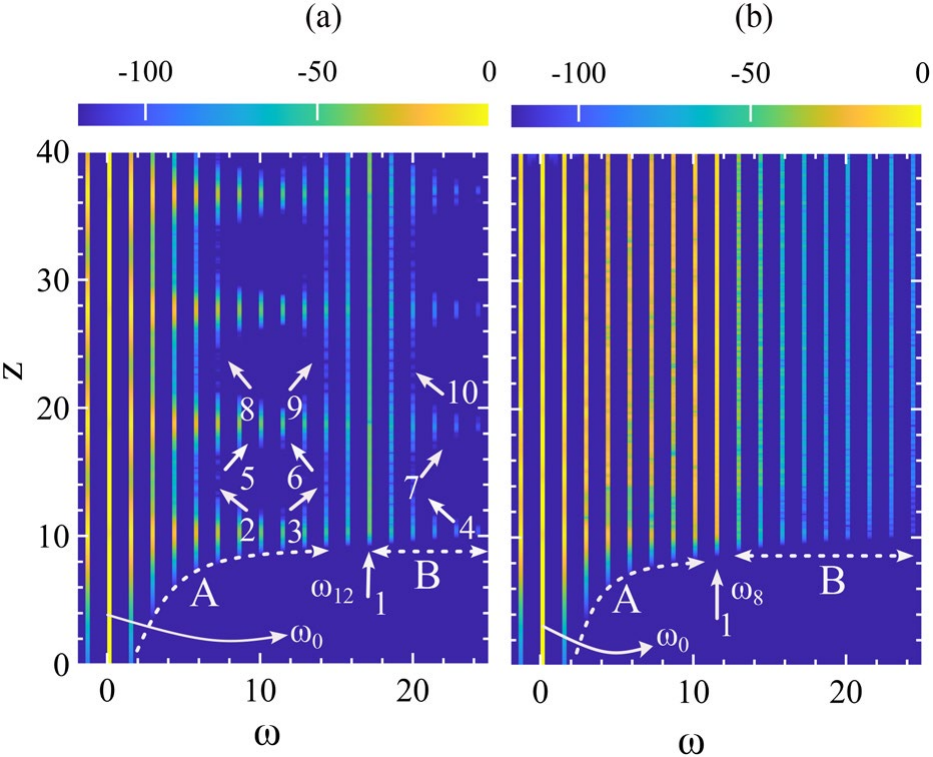
Same as frequency evolution from Fig. 2a, b, the AB’s spectral evolution along *z* with (**a**) $\beta _4 = 0.04$ and (**b**) $\beta _4 = 0.1$ showing the frequency component in $+ \omega$ evolving up to $z=40$. The numbered arrows 2 to 10 show energy flow among the harmonics. The dashed long arrows (A & B) show the range of frequencies on both sides of the phase-matched excited harmonics in (**a**) $\omega _{12}$ and (**b**) $\omega _8$.

In the resonant MI interaction regime, we observed that the presence of FOD with positive $\beta _4$ creates a complex energy exchange scenario during the AB development. In this section, we give a comprehensive explanation of how this happens and its connection to creating a highly complex temporal pattern as shown in Fig. 2b. Note a detailed explanation of the cascaded four-wave mixing process involved in generating this type of discrete harmonics is given in^41^. In this work, considering the experimental scenario two pump frequencies ($\omega _{\pm p}$) on both sides of the central mean frequency are induced to initiate the cascaded four-wave mixing process. However, our narrative is aligned with the work^15^ where the central mean frequency is considered to be the pump where all the energy is stored, and following the four-wave mixing process, the subsequent harmonics are developed. We emphasize more on the directional flow of energy in the AB’s extended evolution dynamics.

Figure 4a is the same as the spectral domain of Fig. 2a. Because the spectrum is symmetric, we highlight only half of it. The AB achieves its first compression point at around $z \approx 10$ while the pump generates and allocates most of its energy into the sidebands along the dashed white arrow indicated by A. The phase-matched harmonic $\omega _{12}$ indicated by the white arrow 1 also acts as a pump and creates sidebands along dashed arrow B. Note that as the pump $\omega _0$ and the harmonic $\omega _{12}$ breathes and exchange energy with the sidebands in a synchronized way, there could be an overlap of shared energy among the sidebands generated by both $\omega _0$ and $\omega _{12}$. In other words, the sidebands generated by $\omega _{12}$ could find their origin in the main pump $\omega _0$.

The AB’s spectrum is widest at its maximum compression point at $z \approx 10$. At the moment, the pump starts to take back its energy again from the sidebands following the rules of Fermi-Pasta-Ulam recurrence^42^. However, instead of flowing all the energy from the sidebands towards the pump $\omega _0$, now the flow is split following arrows 2, 3 and 4. While a part of the energy is flowing towards $\omega _0$ indicated by 2, the remaining energy is flowing towards $\omega _{12}$ shown by the arrows 3 and 4. This marks the end of the first recurrence.

The beginning of the second recurrence starts with the transfer of energy from the pumps to the newly created sidebands indicated by the arrows 5, 6, and 7. At the second compression point, the AB reaches its highest amplitude at $z\approx 19$. Return from this summit to the background is complete when the pump takes back its energy following the arrows 8, 9, and 10. This cycle of energy exchange keeps repeating along *z*. Because energy is flowing from two pumps in the same sidebands between $\omega _0$ and $\omega _{12}$, there is a build-up of energy among the sidebands and this grows with increasing $\beta _4$ values.

**Figure 5:**
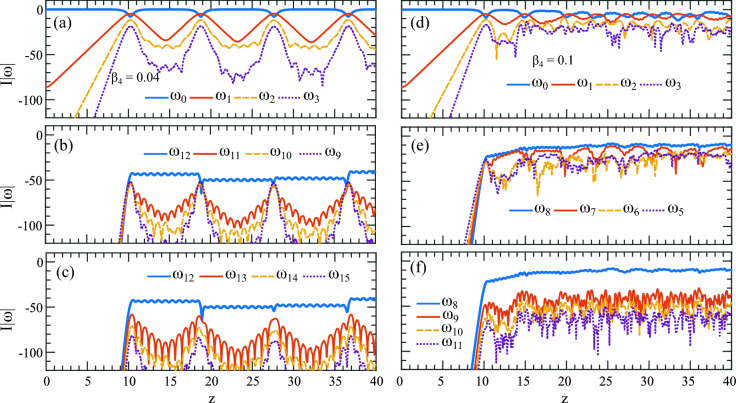
Evolution and energy interaction plots along $z$ for the pump and its next three harmonics from Fig. 4a, b. The top blue curve in all examples represents the pump. (**a**) Pump $\omega _0$ exhibits energy exchange with neighboring sidebands $(\omega _{1},\omega _{2},\omega _{3})$ while maintaining recurrence behavior along $z$. (**b**) The harmonic $\omega _{12}$, excited at the resonant frequency $\omega _{R2} = 17.26$, now acts as a pump (marked by the white arrow in Fig. 4a), interacting with sidebands $(\omega _{11},\omega _{10},\omega _{9})$ originating from its left side. Similarly, (**c**) illustrates the evolution of three sidebands $(\omega _{13},\omega _{14},\omega _{15})$ on the right of $\omega _{12}$. (**d**) Shows the evolution of the first three sidebands $(\omega _{1},\omega _{2},\omega _{3})$ in Fig. 4b next to the pump $\omega _0$ with $\beta _4 = 0.1$, revealing energy exchange. (**e**) Displays the interaction of harmonic $\omega _8$ indicated by the white arrow as the pump with sidebands $(\omega _7, \omega _6, \omega _5)$ situated on its left. Similarly, (**f**) shows the interaction of sidebands $(\omega _9, \omega _{10}, \omega _{11})$ with $\omega _8$ on the right.

This is clarified in Fig. 5 where the spectral intensity is plotted in the longitudinal *z* direction for $\beta _4 = 0.04$. In Fig. 5a, the top-most thick blue line is the pump $\omega _0$ shown in Fig. 4a with an arrow and the curves below are the next three sidebands to its right. The pump depletes at each AB’s compression point by transferring energy to the sidebands. Generally, the nearest few sidebands have the most energy. The further the sidebands are away from the pump, the less energy it acquires. To show the energy exchange, we only plot the next three sidebands from the pumps. Compared with the temporal evolution in Fig. 2a, Fig. 5a clearly shows four AB recurrences with compression and decompression dynamics up to along $z=40$.

In Fig. 5b, c, the top-most thick blue line is the phase-matched harmonic $\omega _{12}$ shown by the arrow 1 in Fig. 5a. Figure 5b shows the energy evolution among three sidebands to the left of $\omega _{12}$ whereas Fig. 5c shows the sidebands to the right. Note that while $\omega _0$ starts to act as a pump from $z=0$, for $\omega _{12}$ it is from $z \approx 10$ after it appears. Because they are pumped by both $\omega _0$ and $\omega _{12}$, the sidebands in Fig. 5b are more amplified and show strong interaction with the pump compared to Fig. 5c. Remarkably, both groups of sidebands $(\omega _{11},\omega _{10},\omega _{9})$ and $(\omega _{13},\omega _{14},\omega _{15})$ interacts with $\omega _{12}$ like regular AB sidebands making this part appeared like a secondary AB as shown in Fig. 2a with a white box. One notable feature is, the pump $\omega _0$ and sidebands in Fig. 5a are far more amplified than in Fig. 5b, c.

A similar observation is made for $\beta _4 = 0.1$ where the sidebands between $\omega _0$ and $\omega _{8}$ are strongly amplified. In Fig. 5d, the topmost thick blue line is the pump $\omega _0$ is indicated by the white arrow 1 in Fig. 4b. The three curves below are the first three harmonics to the left side of $\omega _0$. In Fig. 5e, f, the topmost blue thick line is the harmonic $\omega _8$ and the lines below are the first three harmonics left and right side of $\omega _8$ respectively. The important observation here is, in Fig. 5d, as the MI progressed further along *z*, it rapidly lost its growth-return (recurrence) cycles, leaving no trace of an AB in the evolution scenario. The three sidebands next to the pump ($\omega _1, \omega _2,\omega _3$) are amplified and interact with the pump at its proximity. Along the entire *z*, the energy in the pump appears to remain the same with almost no energy exchange with the sidebands which characteristically indicates the presence of strong linear waves at play. They are dominating the wave dynamics, and the interference among them creates complex temporal patterns.

Figure 5e shows the pump as the resonant harmonic $\omega _8$ which arises at the AB’s first compression point at $z\approx 10$. The first three sidebands to its left are ($\omega _7, \omega _6, \omega _5$). Both groups of sidebands ($\omega _1, \omega _2, \omega _3$) in Fig. 5d and ($\omega _7, \omega _6, \omega _5$) in Fig. 5e are heavily pumped by both $\omega _0$ and $\omega _8$ and the energy is trapped between them resulting in a strong amplification of these sidebands. This amplification is seen in Fig. 4b along the dashed arrow *A* which is as strong as the primary sidebands close to $\omega _0$. However, in this situation, while harmonic $\omega _8$ acts as a pump it also works as a barrier. It prevents energy flow from $\omega _0$ to the sidebands $\omega _9, \omega _{10}, \omega _{11}$ making them less amplified shown in Fig. 5f where the intensity of the harmonics are far below the pump $\omega _8$.

**Figure 6:**
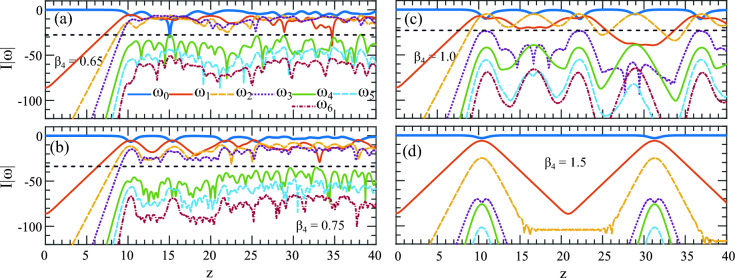
The sideband interactions highlighting the restoration of the growth-return cycle. Evolution of first six harmonics next to pump along *z* for (**a**) $\beta _4 = 0.65$, (**b**) $\beta _4 = 0.75$, (**c**) $\beta _4 = 1.0$ and (**d**) $\beta _4 = 1.5$ of the frequency components evolving up to $z=40$. (**d**) The perfect growth-return dynamics with $\beta _4=1.5$.

In Fig. 6a, with $\beta _4 = 0.65$, we can observe that the pump (blue thick line) is still highly aperiodic. Its energy exchange with the neighboring pumps $\omega _1$ to $\omega _{6}$ is almost static, hence no growth-return cycle leaving no trace of an AB in the *z* direction as outlined and demonstrated in Fig. 2c. With higher values of $\beta _4$, the AB’s spectrum reduced significantly with fewer sideband excitations. We only need to plot the first six harmonics to visualize the energy exchange interactions with the sidebands. These are shown in Fig. 6b–d where the black dashed line separates between more and less amplified sidebands. Starting from $\beta _4=0.75$, we can observe the gradual restoration of an AB’s growth return cycle from the behavior of the pump with increasing $\beta _4$ value. The full restoration is achieved in Fig. 6d with $\beta _4 = 1.5$.

## Evolution trajectory of an AB under the influence of $\beta _4$

**Figure 7:**
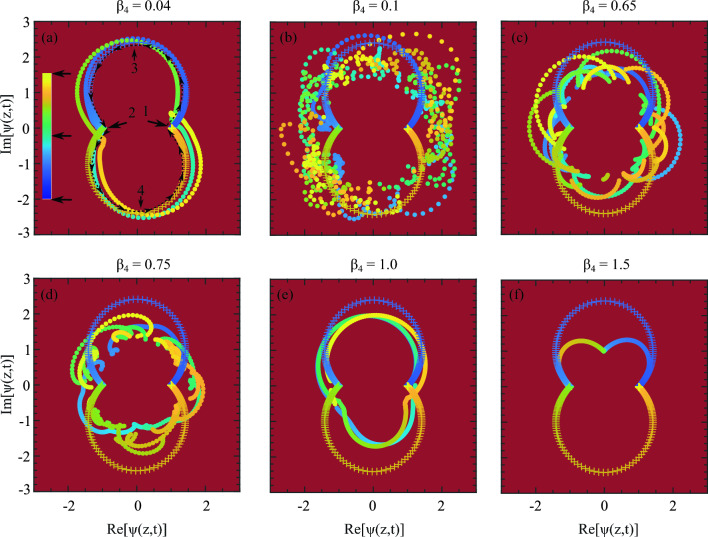
The trajectory of the ABs recurrence cycles on a complex plain. An ideal AB trajectory is presented with marker $+$ in each from (**a**–**f**). The beginning of the AB’s amplitude development starts following the dark-blue which is at the bottom arrow on the colorbar. This point is indicated by the black arrow 1 inside the upper-trajectory loop and the journey ends after one growth-return cycle at the middle arrow on the colorbar. This position is indicated by the black arrow 2 inside the loop. The beginning of the second growth-return cycle starts again at arrow 2 but follows the downward loop and ends at arrow 1 again taking the color bright yellow at the top of the colorbar. The top row shows for (**a**) $\beta _4 = 0.04$, (**b**) $\beta _4 = 0.1$, and (**c**) $\beta _4 = 0.65$ where the bottom row is with (**d**) $\beta _4 = 0.75$, (**e**) $\beta _4 = 1.0$ and (**f**) $\beta _4 = 1.5$. Provided Supplementary Material also highlights these dynamics.

Another way to describe and visualize the MI dynamics is to explore the movement of the AB’s development trajectory on a complex plane. To investigate this we set a parallel where the first row of figures in Fig. 7 corresponds to the temporal evolution of ABs in Fig. 2 with weaker dispersion values. Similarly, the second row corresponds to the AB’s temporal evolution in Fig. 3 with intermediary to stronger dispersion values. Each example of Fig. 7, shows how an AB develops along the evolution direction and undergoes multiple highs and lows creating a specific trajectory of change in a complex plane. The development trajectory of a standard AB followed by the marker $+$ is shown by a black-dotted arrow line. This is rather clearly seen in the last example Fig. 7f and we do this to highlight how $\beta _4$ disrupts an AB’s dynamics when it develops compared to a standard one.

In Fig. 7a, the arrow 1 marks the start of the trajectory (bottom arrow on the colorbar) where ABs amplitude development starts. As the amplitude develops, the change in color profile follows the colorbar in the inset. When AB completes one recurrence, the trajectory ends at arrow 2 (middle arrow on the colorbar) following the upper half of the trajectory. One recurrence means the AB reached its maximum height and came back again on the same background field.

For the next recurrence, the trajectory again begins at arrow 2, that is marking the color profile with the middle arrow in the colorbar and it completes the lower half trajectory marking the ends at the top arrow in the colorbar. With $\beta _4=0$, within evolution length $z=40$, a standard AB appears twice, hence we observe only one upper and a lower trajectory. If there are more recurrences of AB appearances, they overlap with each other. However, these overlaps may not be perfect depending on how irregularly the AB is evolving. The arrows 3 and 4 indicate where the AB reaches its highest amplitude.

With $\beta _4=0.04$ in Fig. 2a, in the temporal evolution, we can see AB appears four times. For this reason, in Fig. 7a, we can see slightly irregular trajectories with round dot markers. However, with increasing $\beta _4$, this trajectory continues to deviate from the ideal path. In Fig. 7b, with $\beta _4 = 0.1$, we observe a drastic change in the ABs development trajectory. In fact, there is hardly any regular path formed which indicates that there is no AB at all. The scattered dots are the peak values of strong background linear waves that show a highly dispersed trajectory indicating the total disappearance of the FPU phenomena and the presence of strong dispersive waves. With further increasing values of $\beta _4 = 0.65$ in Fig. 7c, although the resolution of the trajectories increased, however, they are still highly irregular indicating persistent resonant interaction and the presence of linear dispersive waves on the wave field.

In Fig. 7d with $\beta _4 = 0.75$, many small irregular and incomplete trajectories are formed. However, these irregular behaviors reduced significantly in Fig. 7e with $\beta _4 = 1.0$ indicating that the disappeared AB due to a strong resonant interaction is appearing again. Finally, with $\beta _4 = 1.5$, because there is only one MI frequency, a smooth and consistent trajectory appeared with the resurgence of the AB again. Note that these are the inner small amplitude trajectories which are much smaller compared to the standard AB trajectory.

**Figure 8:**
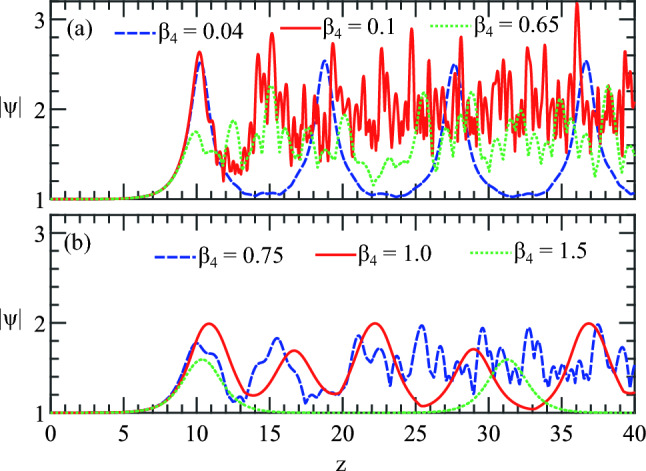
ABs amplitude variation along the propagation distance *z* with (**a**) weak $\beta _4$ and (**b**) strong $\beta _4$. The corresponding $\beta _4$ values are the same as in Fig. 7 (**a**) for the top and (**b**) for the bottom row respectively.

The trajectories in the complex plane can also be complemented by plotting the maxima of the ABs along $z$. The figures in the first and second rows of Fig. 7 correspond to Fig. 8a, b, respectively. In Fig. 8a, due to strong resonant MI interaction, the trails of the temporal peak values exhibit high oscillations due to the presence of amplified dispersive waves.

The blue dashed line, representing $\beta _4=0.04$, still shows regular recurrence dynamics due to the weak values of $\beta _4$; the resonant MI frequencies interaction and the generated dispersive waves are not strong enough to eliminate the AB. However, with $\beta _4 = 0.1$ and $0.65$ in Fig. 8a, the maxima indicated by the green and red dotted lines become highly irregular due to the strong presence of the dispersive wave, and the AB’s recurrence behavior disappears completely. These correspond to Fig. 7b, c in the complex plane presentation.

In Fig. 8b, when the resonance frequency band is closer to or inside the main MI band with $\beta _4 = 0.75$ and $1.0$, the maxima of the ABs fluctuate less, and the recurrence AB dynamics reappear. With $\beta _4 = 1.5$, the green dotted curve shows perfect Fermi–Pasta–Ulam (FPU) recurrence phenomena, corresponding to Fig. 7f with resurgence of the AB. Note, that the maxima developed with weaker $\beta _4$ achieve higher amplitudes compared to those with stronger values.

## Discussion

While previous studies have explored the impact of TOD and FOD on MI, a systematic examination of various MI regimes and their specific influence on AB development has not been addressed^36,43^. Although the disappearance and reappearance of AB with varying strengths of higher-order dispersion have been investigated concerning third-order dispersion^23^, an explanation for this phenomenon was not presented. A more comprehensive overview of various perturbations and robustness of an AB can be found in a recent collection of research topics^44^.

Recently the emergence of sub-regions in the MI band is also reported in vector Manakov equations where the stable gaps between MI bands are discussed^45^. The MI frequency harmonics that fall within these stable gaps do not grow into an AB in both vector and scalar cases. However, the main differences between these two systems are, that the regions of active MI and their shape are different (see Fig. 1b in Ref.^44^ and Fig. 1a in this manuscript). Also, the highest growth rates in sub-MI bands of the vector Manakov systems are unequal whereas in the scalar GNLSE, they remain equal which may play a crucial role in strong resonant interactions and spectral amplification. Another important difference is in the Manakov system, the splitting of the MI band is not related to the higher-order dispersion whereas in our case, it is for the FOD. Indeed it is remarkable to realize that even without higher-order dispersion the Manakov systems allow such resonant interactions. Nonetheless, to reveal the true extent of similarities and differences between these systems requires more in-depth research.

Our goal in this work is to investigate how the introduction of higher-order dispersion in the scalar GNLSE system reveals resonant MI dynamics that significantly affect the extended temporal and spectral evolution of an AB. Also, the role of strong dispersive waves arising from resonant MI on an AB has not been explored. The current study addresses these limitations.

In summary, we utilized the GNLSE and AB solutions to illustrate the influence of FOD on MI dynamics. Under anomalous dispersion conditions, the incorporation of $+\beta _4$ revealed a resonant MI regime and we explained in detail how it impacts the evolution of an AB. Our results demonstrated that FOD introduces complex behaviors in general MI dynamics, drastically affecting AB development in both the temporal and spectral domains. As $\beta _4$ values increase, the resonant MI regimes manifest a complex energy exchange process. We elucidated how this complex energy exchange process amplifies frequencies between the pump and phase-matched harmonics, resulting in a variety of intricate temporal patterns.

Note that while we addressed several key points, our analysis is largely based on the spectral behavior of the interacting ABs. However, it is also important to conduct a comprehensive analysis of the temporal dynamics to answer several crucial questions. One such scenario is how $\beta _4$ is related to varieties of complex patterns that form in the temporal domain. Exceedingly small changes in $\beta _4$ generate entirely different temporal structures (see the Supplementary Material). In the spectral domain, this variation arises only with a narrow or a wide spectral bandwidth. There must be a specific amplitude and phase relationship among the ABs and the background dispersive waves which play a central role in creating those composite patterns. Realizing these connections will enrich our understanding of how the spontaneous emergence of ordered structures forms in nature. Herewith, we acknowledge that AB dynamics under higher-order dispersion and nonlinear effects currently remain a field of active research^17,44^. These open questions may stimulate more debates and discussions leading to more concrete answers.

Our observation and analysis in this work provide a fresh insight into the intricacies of MI dynamics influenced by FOD. Practical applications include optimizing optical parametric amplification processes in diverse waveguide setups. Furthermore, this study contributes to a deeper understanding of MI-induced continuous-wave supercontinuum generation, particularly in CMOS-compatible on-chip waveguides and photonic crystal fibers with substantial nonlinearity and strong dispersion^20,25,26,28^. Our research simplifies the comprehension of MI in these systems, potentially advancing their application in nonlinear light generation and the controlled formation of optical rogue waves. Besides, self-organization is a fascinating phenomenon in the field of nonlinear science. This process plays a pivotal role in shaping spatial patterns in fields such as biology, neural networks in the brain, chemistry, physics, fluid dynamics, and plasmas^7,46^. Current observation of intricate and complex pattern formation highlights the crucial connections between MI and self-organization which may improve our understanding of this highly complex process.

## Methods

The propagation of a modulated continuous wave (CW) inside a waveguide involves employing the generalized nonlinear Schrödinger equation (GNLSE), given by:

7 $$\begin{aligned} i\frac{\partial \psi }{\partial z} -\frac{\beta _2}{2}\frac{\partial ^2 \psi }{\partial t^2}+\gamma \,\, |\psi |^2\psi = i \frac{\beta _3}{6}\frac{\partial ^3 \psi }{\partial t^3}+\frac{\beta _4}{24}\frac{\partial ^4 \psi }{\partial t^4} \end{aligned}$$

It is important to note that $\beta _3$ is omitted in the investigation of MI through linear stability analysis. For phase matching in a four-wave mixing process, the $\beta _3$ term cancels out, as shown in^14^ and^20^ (see Eq. 10.1.7), leading to $\beta _3 = 0$. Although the theory of an AB establishes that odd-order dispersion introduces velocity in the pulse profile while even-order dispersion influences MI and the phase of the pulse profile^47^, it is crucial to clarify that the literature sometimes mistakenly suggests that third-order dispersion (TOD) does not contribute to MI dynamics at all. Contrary to this misconception, several works, such as^22,23,48^, have presented numerical studies on TOD’s contribution to MI.

For numerical simulations, we employed the standard split-step Fourier method in conjunction with the fourth-order Runge–Kutta method to solve Eq. (7)^49^. When $\beta _4 > 0$ and the second term with a positive sign in Eq. (7) is retained, the equation admits a steady-state solution in the form of:

8 $$\begin{aligned} \psi =\sqrt{P_0} \exp (i P_0 \gamma z) \end{aligned}$$

To assess the stability of this solution, the amplitude is perturbed at frequency $\omega$, modifying the steady-state solution to:

9 $$\begin{aligned} \psi =\big [\sqrt{P_0}+a(z,t)\big ] \exp (i P_0 \gamma z) \end{aligned}$$

where $a(z,t) = a_1\, \text {cos}(k z-\omega t)+i a_2\, \text {sin}(kz-\omega t)$. Solving Eq. (7) under these conditions yields the dispersion relation given by:

10 $$\begin{aligned} k =\frac{1}{2}\left( \sqrt{B_2+B_4}\right) \end{aligned}$$

Here, $B_2$ and $B_4$ are defined as follows:

11 $$\begin{aligned} B_2 = \omega ^2 \beta _2^2 \left( \omega ^2+\omega _c^2\right) , \quad B_4 = (\beta _4 \omega ^4/144) \left[ 12 \beta _2 \left( 2 \omega ^2+\omega _c^2\right) +\omega ^4 \beta _4\right] \end{aligned}$$

with $\omega _c^2 = (4\,P_0\,\gamma )/\beta _2$. The dispersion relation in Eq. (10) clearly illustrates the relative influences of the group velocity dispersion parameter $\beta _2$ and the FOD $\beta _4$. The stability of the initial plane wave is determined by the reality of *k*, where instability occurs only when *k* is imaginary. It is essential to highlight that, under specific conditions ($\gamma = P_0 =1$, $\beta _2 = -1$, resulting in $\omega _c^2 = -\,4$), Eq. (10) represents the dispersion relation for the normalized form of Eq. 7^14,32^. This specific form is utilized in our analysis. Furthermore, when $\beta _4=0$, Eq. (10) yields $k=\omega /2\, \sqrt{\left( \omega ^2-4\right) }$, providing the gain expression directly in AB solutions as presented in^15,39^. For $\beta _2 <0$ and $\beta _4 = 0$, $k$ becomes imaginary when $(\beta _2+\beta _4 \, \omega ^2/12) <0$ and

$$\begin{aligned} (\beta _2+\beta _4 \, \omega ^2/12) (\omega ^2/\beta _2) <\omega _c^2 \end{aligned}$$

With these conditions, the gain can be given as^14,20,35^:

12 $$\begin{aligned} g(\omega ) = \text {Im}(k) \end{aligned}$$

### Supplementary Information

Supplementary Video 1.

## Data Availability

The data that support the findings of this study are available from the corresponding author upon reasonable request.
